# Association between Hyperacusis and Tinnitus

**DOI:** 10.3390/jcm9082412

**Published:** 2020-07-28

**Authors:** Christopher R. Cederroth, Alessandra Lugo, Niklas K. Edvall, Andra Lazar, Jose-Antonio Lopez-Escamez, Jan Bulla, Inger Uhlen, Derek J. Hoare, David M. Baguley, Barbara Canlon, Silvano Gallus

**Affiliations:** 1 Laboratory of Experimental Audiology, Department of Physiology and Pharmacology, Karolinska Institutet, 171 77 Stockholm, Sweden; niklas.edvall@ki.se (N.K.E.); barbara.canlon@ki.se (B.C.); 2 National Institute for Health Research (NIHR) Nottingham Biomedical Research Centre, Nottingham University Hospitals NHS Trust, Ropewalk House, Nottingham NG1 5DU, UK; Derek.Hoare@nottingham.ac.uk (D.J.H.); David.Baguley@nottingham.ac.uk (D.M.B.); 3 Hearing Sciences, Division of Clinical Neuroscience, School of Medicine, University of Nottingham, Nottingham NG7 2UH, UK; 4 Department of Environmental Health Sciences, Istituto di Ricerche Farmacologiche Mario Negri IRCCS, 20156 Milan, Italy; alessandra.lugo@marionegri.it (A.L.); silvano.gallus@marionegri.it (S.G.); 5 Hörsel och balansmottagningen, Karolinska Universitetssjukhuset, 171 76 Stockholm, Sweden; andra.lazar@sll.se (A.L.); inger.uhlen@sll.se (I.U.); 6 Otology & Neurotology Group, Department of Genomic Medicine, Pfizer-Universidad de Granada-Junta de Andalucía Centro de Genómica e Investigación Oncológica (GENYO), PTS, Avenida de la Ilustración 114, 18016 Granada, Spain; antonio.lopezescamez@genyo.es; 7 Department of Otolaryngology, Hospital Universitario Virgen de las Nieves, Instituto de Investigacion Biosanitaria ibs.GRANADA, 18012 Granada, Spain; 8 Department of Mathematics, University of Bergen, 5020 Bergen, Norway; Jan.Bulla@uib.no; 9 Department of Psychiatry and Psychotherapy, University of Regensburg, 93053 Regensburg, Germany

**Keywords:** tinnitus, hyperacusis, TMJ, headache, migraine, hearing loss, subtype, somatosensory

## Abstract

Many individuals with tinnitus report experiencing hyperacusis (enhanced sensitivity to sounds). However, estimates of the association between hyperacusis and tinnitus is lacking. Here, we investigate this relationship in a Swedish study. A total of 3645 participants (1984 with tinnitus and 1661 without tinnitus) were enrolled via LifeGene, a study from the general Swedish population, aged 18–90 years, and provided information on socio-demographic characteristics, as well as presence of hyperacusis and its severity. Tinnitus presence and severity were self-reported or assessed using the Tinnitus Handicap Inventory (THI). Phenotypes of tinnitus with (*n* = 1388) or without (*n* = 1044) hyperacusis were also compared. Of 1661 participants without tinnitus, 1098 (66.1%) were women and 563 were men (33.9%), and the mean (SD) age was 45.1 (12.9). Of 1984 participants with tinnitus, 1034 (52.1%) were women and 950 (47.9%) were men, and the mean (SD) age was 47.7 (14.0) years. Hyperacusis was associated with any tinnitus [Odds ratio (OR) 3.51, 95% confidence interval (CI) 2.99–4.13], self-reported severe tinnitus (OR 7.43, 95% CI 5.06–10.9), and THI ≥ 58 (OR 12.1, 95% CI 7.06–20.6). The association with THI ≥ 58 was greater with increasing severity of hyperacusis, the ORs being 8.15 (95% CI 4.68–14.2) for moderate and 77.4 (95% CI 35.0–171.3) for severe hyperacusis. No difference between sexes was observed in the association between hyperacusis and tinnitus. The occurrence of hyperacusis in severe tinnitus is as high as 80%, showing a very tight relationship. Discriminating the pathophysiological mechanisms between the two conditions in cases of severe tinnitus will be challenging, and optimized study designs are necessary to better understand the mechanisms behind the strong relationship between hyperacusis and tinnitus.

## 1. Introduction

Approximately 2% of the population is extremely bothered by tinnitus [1], showing an increased risk in suicide attempts [2,3] and seeking medical support. Patients with severe tinnitus often present themselves with anxiety, depression and stress [4,5,6,7,8], thus largely affecting life quality. There are no effective treatments [9,10,11], and health care costs are substantial [12,13].

The reduced tolerance to general everyday sounds is also known as hyperacusis, in which the sounds are uncomfortably loud or painful, ultimately impairing social, occupational and recreational activities [14]. Experienced by nearly 9% of the population, hyperacusis is more prevalent in people with Williams Syndrome or autism spectrum disorders [15]. Hyperacusis can also be seen in disorders of perception involving the visual and somatosensory functions with higher light sensitivity, headaches and lower pain thresholds in persons with chronic pain [16]. Interestingly, about 90% of people with hyperacusis report concurrent tinnitus, suggesting a strong relationship [17]. While a number of studies investigate the relationship between hyperacusis and tinnitus [18], estimates of the association between tinnitus and hyperacusis in humans is lacking and remains a major research question [19].

Recent studies suggest that tinnitus could emerge as a failure to adapt to missing sensory information from the ear [20]. This loss of sensory input leads to an enhancement of the auditory stimuli in the auditory central system, also known as central neural gain. This condition is characterized by altered tuning bandwidths, increased spontaneous and synchronous neural activity, which may contribute not only to tinnitus, but also to hyperacusis [21]. It has, however, been very difficult to distinguish the neural correlates of tinnitus from those of hyperacusis, in both animal and human studies. In a model of salicylate, in which rats displayed behavioral evidence of both tinnitus and hyperacusis, the auditory network was found to be coupled to the cerebellum, the amygdala and the reticular formation, as evidenced with resting-state functional magnetic resonance imaging (fMRI) [22]. Similar findings were found in humans with tinnitus, but in those studies, hyperacusis was either low or being excluded at recruitment [23,24]. Hyperacusis has been correlated with greater sound-evoked activity in the inferior colliculus (IC), the medial geniculate body (MGB), and the auditory cortex (AC). In a model of active loudness, it has been proposed that hyperacusis results from increased non-linear gain, whereas tinnitus results from central noise independent of gain [25].

Here, we investigate the association between tinnitus and hyperacusis using data from the Swedish Tinnitus Outreach Project and further analyze the phenotypic traits related to tinnitus with accompanying hyperacusis.

## 2. Experimental Section

### 2.1. Design and Ethics

The initial step of this study is an exploratory analysis to determine the association between hyperacusis and tinnitus. This was performed on individuals with or without tinnitus from the Swedish Tinnitus Outreach Project (STOP) who answered to the European School for Interdisciplinary Tinnitus Research Screening Questionnaire (ESIT-SQ). The following step of this work focused only on tinnitus participants with or without hyperacusis in view of characterizing their phenotype. The STOP dataset, participant recruitment via LifeGene [26] and acquisition of consent has been reported previously. Briefly, participants are adults (18 years of age and above) who provided written consent for handling their personal data. The project was approved by the Regional Ethics Review Board in Stockholm (2015/2129-31/1).

### 2.2. Association Study

The ESIT-SQ is a self-report assessment of variables including known or potential risk factors for tinnitus that can be answered by people with or without tinnitus [27]. Question A17 was used to define tinnitus: “Tinnitus refers to the perception of noise in your head or ears (such as ringing or buzzing) in the absence of any corresponding source of sound external to your head. Over the past year, have you had tinnitus in your head or in one or both ears that lasts for more than five minutes at a time?” with answer options being: “do not know”, “no never”, “no, not in the past year”, “yes, some of the time”, “yes a lot of time”, “yes most of the time”. Those answering “don’t know” or “no, not in the past year” were excluded from the analysis. To define hyperacusis in the association study, question A12 was used and stated: “Over the last week, have external sounds been a problem, being too loud or uncomfortable for you when they seemed normal to others around you?” with answer options being: “no, not a problem”, “yes, a small problem”, “yes, a moderate problem”, “yes, a big problem”, “yes, a very big problem”. Those answering “don’t know” were excluded from the analysis. Question A13 was used to define hearing ability: “Do you currently have any other difficulty with your hearing, such as listening to speech in a noisy situation?” with answers “yes, cannot hear at all”, “yes, severe difficulty”, “yes, moderate difficulty”, “yes, a slight difficulty”, “no difficulty”. Those answering “do not know” were excluded. In the Stockholm County, a score ≥ 58 of the Tinnitus Handicap Inventory (THI) is the cut-off for referral to specialty care [28]. We thus used this to infer these participants had high enough severity for referral and subsequent medical diagnosis. Level of education of study participants was categorized in low (no school, primary, lower secondary or upper secondary school) and high (university or higher degree). For the association study, we included 1984 tinnitus cases and 1661 participants without tinnitus from the STOP project.

### 2.3. Tinnitus Phenotyping Study

This part includes all tinnitus participants that answered the ESIT-SQ (n = 1984) and an additional 448 participants with tinnitus that answered a Swedish-validated online survey described elsewhere [29]. Tinnitus was defined as: “Do you have tinnitus?” with the following answers: “do not know”, “no”, “yes, occasionally (now and then)”, “yes, always (all the time)”. All of those with occasional and constant tinnitus were included in this analysis, representing the “any tinnitus” group. The on-line survey included the Tinnitus Sample Case History Questionnaire (TSCHQ) used for the phenotypic characterization of tinnitus patients [30]. Here, question #29 “Do sounds cause you pain or physical discomfort?” was used to define hyperacusis in the phenotyping study, as reported by Schecklemann et al. [31]. Possible answers were: “yes”, “no”, “don’t know”. The intra-class coherent coefficient in a test-retest for the Swedish adaptation of this question is 0.46 (good) [29]. The remaining part of the survey included the Tinnitus Handicap Inventory (THI, Cronbach’s alpha = 0.93) [32,33], the Tinnitus Functional Index (TFI, α = 0.97) [34,35], the Fear of Tinnitus Questionnaire (FTQ, α = 0.71) [36], the Tinnitus Catastrophizing Scale (TCS, α = 0.93) [36], the Hyperacusis Questionnaire (HQ, α = 0.90) [37,38], the Perceived Stress Questionnaire (PSQ, α = 0.94) [39], the Hospital Anxiety Depression Scales for Anxiety (HADS A, α = 0.85) [40] and depression (HADS D, α = 0.83) [40], and the World Health Organization’s Quality of Life (WHOQoL)-BREF [41] was used to determine physical health (α = 0.84), Psychological (α = 0.84), social relationships (α = 0.69), and environment (α = 0.78). The Cronbach’s alpha associated with each questionnaire represents the score of the Swedish adaptation [29]. Numerical Rating Scales (NRS) for from the TSCHQ (questions 12, 16, and 17) allowed us to determine tinnitus loudness, awareness and annoyance.

### 2.4. Statistical Analysis

In the association study, the odds ratios (ORs) and correspondence 95% confidence intervals (CIs) for tinnitus were determined using unconditional multiple logistic regression models using sex, age, educational level and hearing ability for adjustment. Logistic regressions were performed in SAS 9.4 (SAS Institute, Cary, NC, USA).

Regarding the tinnitus phenotyping study, the methods has been described before [42]. Sociodemographic data was obtained using questions from Svensson et al. [43]. For nominal variables, Pearson’s Chi-squared test was used. The non-parametric Wilcoxon’s test was used for all other comparisons. Correction for multiple comparisons was performed using Benjamini and Hochberg test. All statistical analyses were performed in JMP 13 (SAS Institute Inc.) and R (R Core Team, 2019).

## 3. Results

### 3.1. Association Study

We first performed an exploration study in the Swedish Tinnitus Outreach Project (STOP) where we identified 1984 participants with tinnitus (950 [47.9%] male; mean [SD] age, 47.7 [14.0] years) and 1661 without tinnitus (563 [33.9%] male; mean [SD] age, 45.1 [12.9] years). Table 1 shows the basic characteristics of participants without tinnitus, with any tinnitus, with self-reported severe tinnitus, or severe tinnitus based on the THI score (≥58). This cut-off score is used in the Region Stockholm as a criterion for referral for specialty care and used here as a proxy for clinically significant tinnitus [28]. Table 1 displays prevalence of different age groups, sex, distribution of educational attainment and hearing ability. Prevalence of higher education decreased with increasing severity from 82.4% in the never tinnitus group down to 58.6% in the severe tinnitus group, and so was the prevalence of severe hearing abilities (3.4% in the never tinnitus group, up to 46.8% in the THI ≥ 58 group).

Table 2 shows the odds ratios for any, severe tinnitus and THI ≥ 58 according to the same variables as above. For the any tinnitus group, the ORs were 3.51 (95% CI, 3.00–4.11) for moderate difficulty in hearing ability, and 13.5 (95% CI, 9.81–18.5) for severe difficulties. The ORs increased to 5.83 (95% CI, 3.59–9.45) for moderate and 69.4 (39.2–122.8) for severe hearing abilities in the THI ≥ 58 group. Regarding hyperacusis, the ORs were 3.24 (95% CI, 2.75–3.82) for moderate hyperacusis and 9.54 (95% CI, 5.75–15.8) for severe hyperacusis in the any tinnitus group (Table 3). In the self-reported severe tinnitus group, ORs were 5.18 (95% CI, 3.47–7.73) for moderate hyperacusis, and 48.0 (95% CI, 24.7–93.1) for severe hyperacusis. Finally, in the THI ≥ 58 group, the ORs rose to 8.15 (95% CI, 4.68–14.2) and 77.4 (95% CI, 35.0–171.3) in the moderate and severe hyperacusis group, respectively. Severe hyperacusis was found rare in the never tinnitus group (1.1%), but increased to high proportions in the THI ≥ 58 group (41.4%) (Table 3). Stratification by sex did not reveal any sex differences, as the ORs for each tinnitus group were similar between men and women (Table 3 and Table S1). Overall, these results support a strong association between hyperacusis and tinnitus, even more so when tinnitus is severe.

### 3.2. Tinnitus Phenotyping Study

Next, we performed a phenotypic analysis on a larger set of tinnitus participants with (*n* = 1388) or without (*n* = 1044) hyperacusis. Participants with any tinnitus and co-morbid with hyperacusis represented 57% of the total tinnitus sample, increasing to 80% with severe tinnitus (Table S2). The proportion of women increased in the any tinnitus group with hyperacusis (*p* ≤ 0.0001), but this bias was not found with increasing tinnitus severity (Table S2). Logistic regression analyses in Table 3 however show that this is due to greater prevalence of hyperacusis in women without tinnitus, yielding similar ORs between males and females (OR_men_: 3.34, 95% CI, 2.54–4.39; OR_women_: 3.62, 95% CI, 2.95–4.42; Table 3).

With the exception of age, all sociodemographic parameters assessed (i.e., marital status, gross income, education level and employment), were found different between any tinnitus groups with or without hyperacusis. However, as tinnitus increased in severity, none of these variables differed between the two groups. Similarly, when assessing the global impact of tinnitus on tinnitus-related burden using a large set of questionnaires [8,29], all aspects related to stress, anxiety, depression, quality of life, tinnitus distress, loudness, annoyance and awareness were found worse in participants with any tinnitus in presence of hyperacusis (*p* ≤ 0.0001 for all questionnaire scores, Table S3). When assessing severe tinnitus, none of these differed between the groups and only the score from the hyperacusis questionnaire (HQ) was found greater in those self-reporting hyperacusis. These findings are consistent with the reporting of tinnitus being worsened by loud noise or in problems tolerating sounds in participants with hyperacusis, regardless of severity (Tables S4 and S5). However, while many aspects of tinnitus differed between any tinnitus participants with or without hyperacusis (e.g., tinnitus onset, onset related events, tinnitus occurrence, time of day of tinnitus emergence, onset of tinnitus, pulsatility, sound of tinnitus, reduction by sounds, somatosensory influences on tinnitus, impact by sleep, stress or medication, the occurrence in the family and/or contacting a clinician and the number of treatments sought), these did not differ in severe tinnitus. The same was found for auditory aspects (e.g., hearing problems and/or use of hearing aids) and other comorbidities (e.g., headache, temporomandibular joint problems, vertigo, neck pain and/or other pain syndromes), where differences occurred in the any tinnitus group, but not in severe tinnitus. These findings overall suggest that hyperacusis does not contribute to greater tinnitus burden when tinnitus is severe.

## 4. Discussion

Our findings reveal a strong association between tinnitus and hyperacusis. This association peaked when both tinnitus and hyperacusis were perceived as severe, reaching an OR of 77.4 (95% CI, 35.0–171.3) in a fully adjusted model. This tight relationship was also confirmed by the high prevalence of hyperacusis in participants with severe tinnitus (80%). Severely impaired hearing ability, which here relates to the difficulty to understand speech in a noisy environment (a proxy of retrocochlear damage), is strongly associated with severe tinnitus, as evidenced with an OR of 137.6 (95% CI, 62.8–301.2). In the absence of adjustment of this factor in the regression model, the association between severe hyperacusis and severe tinnitus reaches 251.7 (95% CI, 120.4–526.6), demonstrating the important confounding effect of hearing ability in both severe tinnitus and hyperacusis. Indeed, in line with previous research [44], hearing disability was strongly related in our dataset, not only with severe tinnitus, but also with severe hyperacusis (OR, 102.3; 95% CI, 56.9–184.2). This relationship with hearing ability is such that once included in the model, multivariable ORs (adjusted for sex, age, level of education and hearing ability) were found below unity with increasing age. In contrast, crude ORs (without any adjustment) reflect the higher percentage of tinnitus among older compared to younger subjects, with ORs above the unity for subjects aged ≥55 compared to those aged <35. How the audiometric profile impacts on the severity of both tinnitus or hyperacusis would require further investigation; nonetheless, our study outlines a link between hyperacusis and tinnitus that will be influential: as hyperacusis is still less well recognized than tinnitus, despite the fact that these go almost hand in hand as severity increases, our work emphasizes the important need for research programs on both tinnitus and hyperacusis. The direction of this relationship (whether hyperacusis leads to tinnitus, or tinnitus leads to hyperacusis) remains however to be investigated.

Participants with severe tinnitus and severe hyperacusis are characterized by a greater proportion of blast exposure, bilateral tinnitus and familial history of tinnitus, which could help defining a clinical profile for patients with both conditions. Since genetics contribute in the familial transmission of bilateral and severe tinnitus in twins and adoptees [45,46], it is possible that hyperacusis is also influenced by genetic factors. Recent studies in mice suggest that hyperacusis may be related to a form of pain and/or damage sensing mechanism [47,48]. The similarity in brain signatures between chronic pain and tinnitus (and/or hyperacusis) emphasizes the need of investigating the genetic overlap between tinnitus and pain. Furthermore, as recent incentives to biobank tinnitus may lead to interesting mechanistical insights into the pathophysiology of tinnitus [49,50], it will be critical to include information on hyperacusis and its severity. Notably, while the phenotyping study refers to hyperacusis as pain like symptoms, the association study employs another definition of hyperacusis as reduced sound tolerance. These two definitions may consist in two distinct or overlapping categories of hyperacusis. The framework proposed by Tyler et al. (2014) of tinnitus characterized by pain, annoyance, loudness or pain reports the various attributes of hyperacusis that are described by patients [51]. These categories have not been empirically validated as yet, and the extent to which there is a consensus that support this proposal is unknown. Thus, subtyping of hyperacusis remains speculative.

In a study from Schecklmann et al., based on data from the Tinnitus Research Initiative (TRI) database, tinnitus patients with hyperacusis were found younger, displayed higher mental and general distress related to tinnitus, and reported pain disorders and vertigo more frequently than those without hyperacusis [31]. Participants with tinnitus and hyperacusis could more often remark that external noise influenced their tinnitus, which could also more frequently be modulated by head and neck movements [31]. Furthermore, these participants reported their subjective hearing function as being worse than those without hyperacusis. While these factors were also found different in the presence or absence of hyperacusis in the any tinnitus group, none were impacted by hyperacusis in the severe group. This may be due to the fact that the sample size of our severe tinnitus group was smaller than the group used in the TRI (*n* = 1713), or that the THI ≥ 58 group from the STOP study was much more severe than that of the TRI (THI range; TRI: 41.9–53.6 vs. STOP: 72–73.6). However, in spite of the small sample size of our THI ≥ 58 group, we have previously reported differences in somatosensory modulations in these participants with or without temporomandibular joint (TMJ) complaints or headache [42,52], which we could not reveal here being influenced by hyperacusis. It thus appears from this study on hyperacusis and our previous reports on TMJ and headache, that with greater tinnitus severity, fewer differences are found with or without the co-morbidities.

Our study suggests that estimates of an association between tinnitus and another condition may be strongly underestimated when working with a broad definition of tinnitus such as the “any” tinnitus presented here, encompassing occasional and constant forms, various levels of severity and duration. Instead, the severe tinnitus group, in which prevalence is close to that of the clinically relevant tinnitus, may represent the appropriate (and more homogenous) target group to focus research efforts on whether in cross-sectional, longitudinal or case/control studies. A limitation originating from this is the low prevalence of severe tinnitus, thus requiring large datasets to compute risks. In addition, given the growing importance of studying the impacts of sex on disease, stratified analyses will reduce the power of such studies, more so when considering subtypes. For instance, only one man without tinnitus reported severe hyperacusis leading to large confidence intervals. Thus, depending on the research question, datasets 10 to 100 times bigger than STOP may offer the possibility of addressing the epidemiology of severe tinnitus.

The inclusion of non-tinnitus controls is also an important contribution from our dataset. Without this group, one would believe that there is an increased risk for hyperacusis in women with tinnitus, whereas the inclusion of non-tinnitus controls shows this increased prevalence of hyperacusis in women already persists in absence of tinnitus, ultimately leading to equal ORs for tinnitus in men and women when having hyperacusis. Since temporomandibular joint disorders and headaches follow a similar pattern, it is possible that these co-morbidities do not contribute to sex differences in the burden associated with tinnitus.

**Figure 1:**
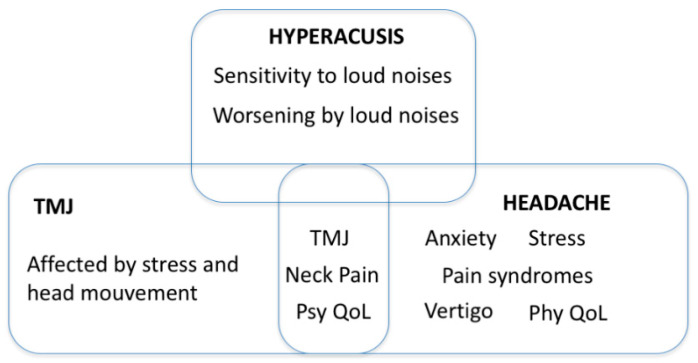
Phenotypes from tinnitus participants with hyperacusis are distinct from those with headaches or temporomandibular joint complaints. Venn Diagram describing the variables from participants with severe tinnitus (THI ≥ 58) that differ between those having TMJ complaints or not [42], those reporting headache or not [52], or hyperacusis or not (the present study). TMJ complaints, neck pain and psychological quality of life (Psy QoL) are found common to TMJ complaints and headache, whereas hyperacusis does not share any of the features appearing in participants with TMJ complaints or headache.

Assuming the severe tinnitus group is the most relevant group to focus tinnitus research on, we may use it to identify co-morbidities shared or distinct between individuals with hyperacusis, headache or TMJ problems. Unlike the previous studies using data from the TRI, where tinnitus with hyperacusis was found comorbid with vertigo, neck pain and TMJ [31], similar to tinnitus with headaches [53], our data indicates that this is not the case (Figure 1). Our results instead suggest that the general assumption that hyperacusis and headaches share similar somatosensory components is wrong. However, the somatosensory components may be common to tinnitus with TMJ complaints or headaches, both of which share neck pain and an impact on psychological quality of life. Hyperacusis, however, stands out with greater sensitivity and worsening of tinnitus by loud noises, something which is not seen in tinnitus with headaches or TMJ complains. Thus, we propose that while tinnitus with TMJ or headaches may share common mechanisms, hyperacusis is distinct, and shows no links to such somatosensory components (neck pain, vertigo and TMJ complains). The additive or synergistic contributions to tinnitus severity by hyperacusis and TMJ/headache remain to be investigated.

### 4.1. Implications for Diagnosis and Treatment

The combined occurrence of tinnitus and hyperacusis, particularly evident when both are severe, has some important implications for diagnosis and treatment [54]. The assessment of both tinnitus and hyperacusis is essential in this population. However, some potential measurements for assessment may be off-limits due to their loud nature (e.g., (f)MRI, auditory brainstem responses, mismatch negativity or gap pre-pulse inhibition of startle response) [55,56,57]. The potential use of hearing aids to reduce the starkness of tinnitus may be restricted by the presence of hyperacusis. Both tinnitus and hyperacusis may be accompanied by anxiety and distress, and these emotional aspects may be compounded by the presence of both symptoms. The determination from the patient of which symptom arose first, and which is their prime complaint could be used in the formulation of a treatment plan. From our data, the benefits of somatosensory [58] or combined auditory-somatosensory [59] treatments would appear to be limited in patients with severe tinnitus and hyperacusis in combination, which further highlights the importance of monitoring the two.

### 4.2. Limitations

First, sample size significantly reduces as both the severity of tinnitus and hyperacusis increase. This could cause bias in the estimates, as for instance only one individual out of 563 men was found with severe hyperacusis in the non-tinnitus group. Thus, larger studies will be required to confirm the strong association between severe tinnitus and severe hyperacusis. Second, both tinnitus and hyperacusis were assessed at one single sample point and therefore the direction of the association remains to be determined. Finally, this study was based on the recruiting of participants from LifeGene, which may pre-empt its generalization to the general population.

## 5. Conclusions

The present study suggests that hyperacusis is strongly associated with tinnitus, and that this relationship increases with severity. Longitudinal studies will help in determining the direction of this association. Unlike in the cases of co-morbid headaches or temporomandibular joint disorders, severe tinnitus with hyperacusis is not accompanied with neck or other pain syndromes, or vertigo and suggests the lack of involvement of trigeminal nerve dysfunctions in the pathophysiology of tinnitus with hyperacusis. Future studies investigating the additive or synergistic interactions of headache and hyperacusis into the severity of tinnitus may provide additional insights into the multifactorial contribution to tinnitus burden.

## Figures and Tables

**Table 1:** Distribution of 1661 participants without tinnitus and 1984 participants with tinnitus ^a^ (157 with a severe tinnitus measured by THI, and 239 with a self-reported severe tinnitus), according to sex, age, level of education, and hearing ability. Sweden, 2016–2018.

	No Tinnitus	Participants with Tinnitus		
		Any Tinnitus	Self-Reported Severe Tinnitus	Severe Tinnitus (THI ≥ 58)
	*n* (%)	*n* (%)	*n* (%)	*n* (%)
Total	1661 (100.0)	1984 (100.0)	239 (100.0)	157 (100.0)
				
Sex ^b^				
Men	563 (33.9)	947 (47.8)	112 (47.1)	71 (45.5)
Women	1098 (66.1)	1034 (52.2)	126 (52.9)	85 (54.5)
Age group (years) ^b^				
<35	330 (19.9)	362 (18.3)	43 (18.0)	37 (23.6)
35–44	478 (28.8)	437 (22.1)	43 (18.0)	26 (16.6)
45–54	459 (27.7)	530 (26.8)	57 (23.9)	36 (22.9)
55–64	197 (11.9)	306 (15.5)	53 (22.2)	32 (20.4)
65–74	160 (9.6)	274 (13.8)	30 (12.6)	20 (12.7)
≥75	35 (2.1)	72 (3.6)	13 (5.4)	6 (3.8)
Level of education				
Low	292 (17.6)	545 (27.5)	99 (41.4)	73 (46.5)
High	1369 (82.4)	1439 (72.5)	140 (58.6)	84 (53.5)
Hearing ability ^b^				
No difficulty	820 (50.1)	362 (18.6)	21 (8.8)	9 (5.8)
Moderate difficulty	761 (46.5)	1233 (63.4)	117 (49.2)	74 (47.4)
Severe difficulty	55 (3.4)	350 (18.0)	100 (42.0)	73 (46.8)

THI: Tinnitus Handicap Inventory. ^a^ 337 participants reporting to have had tinnitus only before the last 12 months were excluded. ^b^ The sum does not add up to the total because of some missing values.

**Table 2:** Odds ratios (OR) ^a^ for any tinnitus, severe tinnitus (THI ≥ 58), and self-reported severe tinnitus, and corresponding 95% confidence intervals (CI), according to sex, age, level of education, and hearing ability. Sweden, 2016–2018.

	Any Tinnitus	Severe Tinnitus (Self-Reported)	Severe Tinnitus (THI ≥ 58)
Sex			
Men	Reference	Reference	Reference
Women	**0.55 (0.48–0.64)**	**0.54 (0.39–0.75)**	**0.59 (0.40–0.88)**
Age group (years)			
<35	Reference	Reference	Reference
35–44	**0.80 (0.64–0.99)**	**0.51 (0.30–0.84)**	**0.30 (0.16–0.56)**
45–54	0.84 (0.67–1.04)	**0.55 (0.34–0.89)**	**0.41 (0.23–0.72)**
55–64	1.05 (0.81–1.36)	**0.82 (0.48–1.40)**	**0.50 (0.26–0.95)**
65–74	1.12 (0.86–1.47)	**0.65 (0.36–1.18)**	**0.46 (0.23–0.93)**
≥75	0.98 (0.61–1.58)	**0.69 (0.28–1.68)**	**0.24 (0.08–0.79)**
p for trend	0.153		0.064
Level of education			
Low	Reference	Reference	Reference
High	**0.68 (0.57–0.81)**	**0.33 (0.23–0.47)**	**0.24 (0.16–0.37)**
Hearing ability			
No difficulty	Reference	Reference	Reference
Moderate difficulty	**3.51 (3.00–4.11)**	**5.83 (3.59–9.45)**	**8.76 (4.31–17.8)**
Severe difficulty	**13.5 (9.81–18.5)**	**69.4 (39.2–122.8)**	**137.6 (62.8–301.2)**

THI: Tinnitus Handicap Inventory. ^a^ ORs were estimated using unconditional multiple logistic regression models after adjustment for sex (men or women), age (<35, 35–44, 45–54, 55–64, 65–74, or ≥75 years), level of education (low or high), and hearing ability (yes, cannot hear at all; yes, severe difficulty; yes, moderate difficulty; yes, a slight difficulty; no difficulty). Estimates in bold are statistically significant at 0.05 level.

**Table 3:** Odds ratios (OR)^a^ for any tinnitus, severe tinnitus (THI ≥ 58), and self-reported severe tinnitus, and corresponding 95% confidence intervals (CI), according to hyperacusis. Sweden, 2016–2018.

	No Tinnitus	Any Tinnitus		Severe Tinnitus (Self-Reported)		Severe Tinnitus (THI ≥ 58)	
	*n* (%)	*n* (%)	OR(95% CI)	*n* (%)	OR (95% CI)	*n* (%)	OR (95% CI)
Both sexes							
Total	1661 (100.0)	1984 (100.0)	-	239 (100.0)	-	157 (100.0)	-
Hyperacusis							
No	1255 (75.6)	822 (41.4)	Reference	51 (21.3)	Reference	21 (13.4)	Reference
Yes	406 (24.4)	1162 (58.6)	**3.51** **(2.99–4.13)**	188 (78.7)	**7.43** **(5.06–10.9)**	136 (86.6)	**12.1** **(7.06–20.6)**
Moderate	387 (23.3)	970 (48.9)	**3.24** **(2.75–3.82)**	105 (43.9)	**5.18** **(3.47–7.73)**	71 (45.2)	**8.15** **(4.68–14.2)**
Severe	19 (1.1)	192 (9.7)	**9.54** **(5.75–15.8)**	83 (34.7)	**48.0** **(24.7–93.3)**	65 (41.4)	**77.4** **(35.0–171.3)**
Males							
Total	563 (100.0)	947 (100.0)	-	112 (100.0)	-	71 (100.0)	-
Hyperacusis							
No	463 (82.2)	467 (49.3)	Reference	31 (27.7)	Reference	13 (18.3)	Reference
Yes	100 (17.8)	480 (50.7)	**3.34** **(2.54–4.39)**	81 (72.3)	**6.88** **(4.03–11.7)**	58 (81.7)	**10.6** **(5.15–22.0)**
Moderate	99 (17.6)	412 (43.5)	**3.03** **(2–30–3.99)**	44 (39.3)	**4.23** **(2.38–7.51)**	34 (47.9)	**7.26** **(3.40–15.5)**
Severe	1 (0.2)	68 (7.2)	**37.0** **(5.03–271.4)**	37 (33.0)	**283.8** **(35.1–>>)**	24 (33.8)	**346.0** **(35.6–>>)**
Females							
Total	1098 (100.0)	1034 (100.0)	-	126 (100.0)	-	85 (100.0)	-
Hyperacusis							
No	792 (72.1)	355 (34.3)	Reference	20 (15.9)	Reference	8 (9.4)	Reference
Yes	306 (27.9)	679 (65.7)	**3.62** **(2.95–4.42)**	106 (84.1)	**7.82** **(4.44–13.8)**	77 (90.6)	**15.3** **(6.49–35.8)**
Moderate	288 (26.2)	556 (53.8)	**3.37** **(2.74–4.14)**	61 (48.4)	**5.88** **(3.29–10.5)**	37 (43.5)	**9.79** **(4.11–23.3)**
Severe	18 (1.6)	123 (11.9)	**7.69** **(4.47–13.3)**	45 (35.7)	**31.6** **(13.7–72.9)**	40 (47.1)	**71.1** **(24.7–204.9)**

THI: Tinnitus Handicap Inventory. ^a^ ORs were estimated using unconditional multiple logistic regression models after adjustment for sex (men or women), age (<35, 35–44, 45–54, 55–64, 65–74, or ≥75 years), level of education (low or high), and hearing ability (yes, cannot hear at all; yes, severe difficulty; yes, moderate difficulty; yes, a slight difficulty; no difficulty). Estimates in bold are statistically significant at 0.05 level. >> refers to values above 999.

## Supplementary Materials

The following are available online at https://www.mdpi.com/2077-0383/9/8/2412/s1, Table S1: Sex stratified analysis of sociodemographics, Table S2: Sociodemographic and psychological characteristics for participants with tinnitus with or without hyperacusis, Table S3: Questionnaire scores from participants with tinnitus with or without hyperacusis, Table S4: Phenotypic characteristics for participants with tinnitus with or without hyperacusis, Table S5: Comorbidities in participants with tinnitus with or without hyperacusis.

## References

[1] McCormack A., Edmondson-Jones M., Somerset S., Hall D. (2016). A systematic review of the reporting of tinnitus prevalence and severity. Hear. Res..

[2] Lugo A., Trpchevska N., Liu X., Biswas R., Magnusson C., Gallus S., Cederroth C.R. (2019). Sex-Specific Association of Tinnitus with Suicide Attempts. JAMA Otolaryngol. Head Neck Surg..

[3] Seo J.H., Kang J.M., Hwang S.H., Han K.D., Joo Y.H. (2016). Relationship between tinnitus and suicidal behaviour in Korean men and women: A cross-sectional study. Clin. Otolaryngol..

[4] Dobie R.A. (2003). Depression and tinnitus. Otolaryngol. Clin. N. Am..

[5] Bartels H., Middel B.L., van der Laan B.F., Staal M.J., Albers F.W. (2008). The additive effect of co-occurring anxiety and depression on health status, quality of life and coping strategies in help-seeking tinnitus sufferers. Ear Hear..

[6] Kehrle H.M., Sampaio A.L., Granjeiro R.C., de Oliveira T.S., Oliveira C.A. (2016). Tinnitus Annoyance in Normal-Hearing Individuals: Correlation with Depression and Anxiety. Ann. Otol. Rhinol. Laryngol..

[7] Hebert S., Canlon B., Hasson D. (2012). Emotional exhaustion as a predictor of tinnitus. Psychother. Psychosom..

[8] Schlee W., Hall D., Edvall N.K., Langguth B., Canlon B., Cederroth C.R. (2017). Visualization of Global Disease Burden for the Optimization of Patient Management and Treatment. Front. Med..

[9] Cederroth C.R., Canlon B., Langguth B. (2013). Hearing loss and tinnitus—Are funders and industry listening?. Nat. Biotechnol..

[10] Cederroth C.R., Dyhrfjeld-Johnsen J., Langguth B. (2018). An update: Emerging drugs for tinnitus. Expert Opin. Emerg. Drugs.

[11] Langguth B., Elgoyhen A.B., Cederroth C.R. (2019). Therapeutic Approaches to the Treatment of Tinnitus. Annu. Rev. Pharmacol. Toxicol..

[12] Stockdale D., McFerran D., Brazier P., Pritchard C., Kay T., Dowrick C., Hoare D.J. (2017). An economic evaluation of the healthcare cost of tinnitus management in the UK. BMC Health Serv. Res..

[13] Maes I.H., Cima R.F., Vlaeyen J.W., Anteunis L.J., Joore M.A. (2013). Tinnitus: A cost study. Ear Hear..

[14] Aazh H., Moore B.C., Lammaing K., Cropley M. (2016). Tinnitus and hyperacusis therapy in a UK National Health Service audiology department: Patients’ evaluations of the effectiveness of treatments. Int. J. Audiol..

[15] Thabet E.M., Zaghloul H.S. (2013). Auditory profile and high resolution CT scan in autism spectrum disorders children with auditory hypersensitivity. Eur. Arch. Oto-Rhino-Laryngol..

[16] Ambrosini A., Schoenen J. (2006). Electrophysiological response patterns of primary sensory cortices in migraine. J. Headache Pain.

[17] Aazh H., McFerran D., Salvi R., Prasher D., Jastreboff M., Jastreboff P. (2014). Insights from the First International Conference on Hyperacusis: Causes, evaluation, diagnosis and treatment. Noise Health.

[18] Baguley D.M., Fagelson M.A., Baguley D.M. (2018). The Epidemiology and Natural History of Disorders of Sound Perception. Hyperacusis and Disorders of Sound Intolerance.

[19] Baguley D.M., Hoare D.J. (2018). Hyperacusis: Major research questions. Hno.

[20] Shore S.E., Roberts L.E., Langguth B. (2016). Maladaptive plasticity in tinnitus—Triggers, mechanisms and treatment. Nat. Rev. Neurol..

[21] Roberts L.E., Salvi R. (2019). Overview: Hearing loss, tinnitus, hyperacusis, and the role of central gain. Neuroscience.

[22] Chen Y.C., Li X., Liu L., Wang J., Lu C.Q., Yang M., Jiao Y., Zang F.C., Radziwon K., Chen G.D. (2015). Tinnitus and hyperacusis involve hyperactivity and enhanced connectivity in auditory-limbic-arousal-cerebellar network. eLife.

[23] Leaver A.M., Renier L., Chevillet M.A., Morgan S., Kim H.J., Rauschecker J.P. (2011). Dysregulation of limbic and auditory networks in tinnitus. Neuron.

[24] Maudoux A., Lefebvre P., Cabay J.E., Demertzi A., Vanhaudenhuyse A., Laureys S., Soddu A. (2012). Auditory resting-state network connectivity in tinnitus: A functional MRI study. PLoS ONE.

[25] Zeng F.G. (2013). An active loudness model suggesting tinnitus as increased central noise and hyperacusis as increased nonlinear gain. Hear. Res..

[26] Almqvist C., Adami H.O., Franks P.W., Groop L., Ingelsson E., Kere J., Lissner L., Litton J.E., Maeurer M., Michaelsson K. (2011). LifeGene—A large prospective population-based study of global relevance. Eur. J. Epidemiol..

[27] Genitsaridi E., Partyka M., Gallus S., Lopez-Escamez J.A., Schecklmann M., Mielczarek M., Trpchevska N., Santacruz J.L., Schoisswohl S., Riha C. (2019). Standardised profiling for tinnitus research: The European School for Interdisciplinary Tinnitus Research Screening Questionnaire (ESIT-SQ). Hear. Res..

[28] Idrizbegovic E., Kjerulf E., Adults T. (2011). Tinnitus Care Program [Tinnitus Vårdprogram].

[29] Müller K., Edvall N.K., Idrizbegovic E., Huhn R., Cima R., Persson V., Leineweber C., Westerlund H., Langguth B., Schlee W. (2016). Validation of Online Versions of Tinnitus Questionnaires Translated into Swedish. Front. Aging Neurosci..

[30] Landgrebe M., Zeman F., Koller M., Eberl Y., Mohr M., Reiter J., Staudinger S., Hajak G., Langguth B. (2010). The Tinnitus Research Initiative (TRI) database: A new approach for delineation of tinnitus subtypes and generation of predictors for treatment outcome. BMC Med. Inform. Decis. Mak..

[31] Schecklmann M., Landgrebe M., Langguth B., TRI Database Study Group (2014). Phenotypic characteristics of hyperacusis in tinnitus. PLoS ONE.

[32] Newman C.W., Jacobson G.P., Spitzer J.B. (1996). Development of the Tinnitus Handicap Inventory. Arch. Otolaryngol. Head Neck Surg..

[33] Newman C.W., Sandridge S.A., Jacobson G.P. (1998). Psychometric adequacy of the Tinnitus Handicap Inventory (THI) for evaluating treatment outcome. J. Am. Acad. Audiol..

[34] Meikle M.B., Henry J.A., Griest S.E., Stewart B.J., Abrams H.B., McArdle R., Myers P.J., Newman C.W., Sandridge S., Turk D.C. (2012). The tinnitus functional index: Development of a new clinical measure for chronic, intrusive tinnitus. Ear Hear..

[35] Henry J.A., Griest S., Thielman E., McMillan G., Kaelin C., Carlson K.F. (2016). Tinnitus Functional Index: Development, validation, outcomes research, and clinical application. Hear. Res..

[36] Cima R.F., Crombez G., Vlaeyen J.W. (2011). Catastrophizing and fear of tinnitus predict quality of life in patients with chronic tinnitus. Ear Hear..

[37] Khalfa S., Dubal S., Veuillet E., Perez-Diaz F., Jouvent R., Collet L. (2002). Psychometric normalization of a hyperacusis questionnaire. ORL J. Oto-Rhino-Laryngol. Its Relat. Spec..

[38] Fackrell K., Fearnley C., Hoare D.J., Sereda M. (2015). Hyperacusis Questionnaire as a Tool for Measuring Hypersensitivity to Sound in a Tinnitus Research Population. BioMed Res. Int..

[39] Levenstein S., Prantera C., Varvo V., Scribano M.L., Berto E., Luzi C., Andreoli A. (1993). Development of the Perceived Stress Questionnaire: A new tool for psychosomatic research. J. Psychosom. Res..

[40] Andersson G., Kaldo-Sandstrom V., Strom L., Stromgren T. (2003). Internet administration of the Hospital Anxiety and Depression Scale in a sample of tinnitus patients. J. Psychosom. Res..

[41] The WHOQOL Group (1998). Development of the World Health Organization WHOQOL-BREF quality of life assessment. Psychol. Med..

[42] Edvall N.K., Gunan E., Genitsaridi E., Lazar A., Mehraei G., Billing M., Tullberg M., Bulla J., Whitton J., Canlon B. (2019). Impact of Temporomandibular Joint Complaints on Tinnitus-Related Distress. Front. Neurosci..

[43] Svensson A.C., Fredlund P., Laflamme L., Hallqvist J., Alfredsson L., Ekbom A., Feychting M., Forsberg B., Pedersen N.L., Vagero D. (2013). Cohort profile: The Stockholm Public Health Cohort. Int. J. Epidemiol..

[44] Nelson J.J., Chen K. (2004). The relationship of tinnitus, hyperacusis, and hearing loss. Ear Nose Throat J..

[45] Cederroth C.R., PirouziFard M., Trpchevska N., Idrizbegovic E., Canlon B., Sundquist J., Sundquist K., Zoller B. (2019). Association of Genetic vs Environmental Factors in Swedish Adoptees with Clinically Significant Tinnitus. JAMA Otolaryngol. Head Neck Surg..

[46] Maas I.L., Bruggemann P., Requena T., Bulla J., Edvall N.K., Hjelmborg J.V.B., Szczepek A.J., Canlon B., Mazurek B., Lopez-Escamez J.A. (2017). Genetic susceptibility to bilateral tinnitus in a Swedish twin cohort. Genet. Med. Off. J. Am. Coll. Med. Genet..

[47] Liu C., Glowatzki E., Fuchs P.A. (2015). Unmyelinated type II afferent neurons report cochlear damage. Proc. Natl. Acad. Sci. USA.

[48] Flores E.N., Duggan A., Madathany T., Hogan A.K., Marquez F.G., Kumar G., Seal R.P., Edwards R.H., Liberman M.C., Garcia-Anoveros J. (2015). A non-canonical pathway from cochlea to brain signals tissue-damaging noise. Curr. Biol..

[49] Szczepek A.J., Frejo L., Vona B., Trpchevska N., Cederroth C.R., Caria H., Lopez-Escamez J.A. (2019). Recommendations on Collecting and Storing Samples for Genetic Studies in Hearing and Tinnitus Research. Ear Hear..

[50] Cederroth C.R., Kähler A., Sullivan P.F., Lopez-Escamez J.A. (2017). Genetics of tinnitus: Time to biobank phantom sounds. Front. Genet..

[51] Tyler R.S., Pienkowski M., Roncancio E.R., Jun H.J., Brozoski T., Dauman N., Dauman N., Andersson G., Keiner A.J., Cacace A.T. (2014). A review of hyperacusis and future directions: Part I. Definitions and manifestations. Am. J. Audiol..

[52] Lugo A., Edvall N.K., Lazar A., Mehraei G., Lopez-Escamez J.A., Bulla J., Uhlén I., Canlon B., Gallus S., Cederroth C.R. (2020). Relationship between headaches and tinnitus in a Swedish study. Sci. Rep..

[53] Langguth B., Hund V., Landgrebe M., Schecklmann M. (2017). Tinnitus Patients with Comorbid Headaches: The Influence of Headache Type and Laterality on Tinnitus Characteristics. Front. Neurol..

[54] Fackrell K., Stratmann L., Gronlund T.A., Hoare D.J. (2019). Top ten hyperacusis research priorities in the UK. Lancet.

[55] Sedley W., Alter K., Gander P.E., Berger J., Griffiths T.D. (2019). Exposing Pathological Sensory Predictions in Tinnitus Using Auditory Intensity Deviant Evoked Responses. J. Neurosci. Off. J. Soc. Neurosci..

[56] Fournier P., Hebert S. (2013). Gap detection deficits in humans with tinnitus as assessed with the acoustic startle paradigm: Does tinnitus fill in the gap?. Hear. Res..

[57] Milloy V., Fournier P., Benoit D., Norena A., Koravand A. (2017). Auditory Brainstem Responses in Tinnitus: A Review of Who, How, and What?. Front. Aging Neurosci..

[58] Van der Wal A., Michiels S., Van de Heyning P., Braem M., Visscher C., Topsakal V., Gilles A., Jacquemin L., Van Rompaey V., De Hertogh W. (2020). Treatment of Somatosensory Tinnitus: A Randomized Controlled Trial Studying the Effect of Orofacial Treatment as Part of a Multidisciplinary Program. J. Clin. Med..

[59] Roberts L.E., Sanchez T.G., Bruce I.C., Fagelscon M., Baguley D.M. (2018). Tinnitus and hyperacusis: Relationship, mechanisms, and initiating conditions. Hyperacusis and Disorders of Sound Intolerance.

[60] Schlee W., Hall D.A., Canlon B., Cima R.F.F., de Kleine E., Hauck F., Huber A., Gallus S., Kleinjung T., Kypraios T. (2017). Innovations in Doctoral Training and Research on Tinnitus: The European School on Interdisciplinary Tinnitus Research (ESIT) Perspective. Front. Aging Neurosci..

