# Supplementary material for: Association between Hyperacusis and Tinnitus
Source: J Clin Med. 2020 Jul 28;9(8):2412. doi: 10.3390/jcm9082412 (PMC7465629; doi:10.3390/jcm9082412)
Supplement: Supplementary file 1 [file jcm-09-02412-s001.pdf]

Table S1. **Sex stratified analysis of sociodemographics**. Odds ratios (OR) [a] for any tinnitus, severe tinnitus (THI≥58), and self-reported severe tinnitus, and corresponding 95% confidence intervals (CI), by sex according to age, level of education, and hearing ability. Sweden, 2016-2018.

	**Any tinnitus**		**Severe tinnitus (self-reported)**		**Severe tinnitus (THI ≥58)**	
	**Men**	**Women**	**Men**	**Women**	**Men**	**Women**
Age group (years)						
<35	1 [Reference]	1 [Reference]	1 [Reference]	1 [Reference]	1 [Reference]	1 [Reference]
35-44	0.84 (0.60-1.19)	0.77 (0.58-1.03)	**0.41 (0.20-0.84)**	**0.62 (0.29-1.33)**	**0.22 (0.09-0.56)**	**0.38 (0.15-0.98)**
45-54	0.73 (0.52-1.03)	0.93 (0.70-1.22)	**0.32 (0.16-0.66)**	0.91 (0.46-1.81)	**0.27 (0.11-0.62)**	0.64 (0.29-1.42)
55-64	1.00 (0.65-1.55)	1.09 (0.79-1.51)	0.51 (0.22-1.19)	1.16 (0.56-2.42)	**0.24 (0.08-0.71)**	0.77 (0.33-1.81)
65-74	0.89 (0.58-1.39)	1.30 (0.93-1.82)	**0.37 (0.15-0.91)**	1.17 (0.52-2.62)	**0.32 (0.11-0.90)**	0.76 (0.29-1.98)
≥75	0.78 (0.40-1.55)	1.23 (0.65-2.36)	0.31 (0.08-1.14)	1.89 (0.58-6.17)	**0.07 (0.01-0.50)**	0.98 (0.22-4.37)
p for trend	0.652	**0.023**	**0.050**	0.128	**0.009**	0.760
Level of education						
Low	1 [Reference]	1 [Reference]	1 [Reference]	1 [Reference]	1 [Reference]	1 [Reference]
High	**0.51 (0.39-0.68)**	0.83 (0.66-1.05)	**0.22 (0.13-0.37)**	**0.49 (0.30-0.81)**	**0.13 (0.07-0.25)**	**0.42 (0.23-0.75)**
Hearing ability						
No difficulty	1 [Reference]	1 [Reference]	1 [Reference]	1 [Reference]	1 [Reference]	1 [Reference]
Moderate difficulty	**3.65 (2.85-4.66)**	**3.45 (2.81-4.23)**	**4.65 (2.46-8.78)**	**8.34 (3.75-18.5)**	**5.67 (2.38-13.5)**	**19.4 (4.62-81.1)**
Severe difficulty	**9.89 (6.02-16.3)**	**16.0 (10.6-24.0)**	**40.7 (17.6-94.0)**	**125.8 (52.7-300.1)**	**70.0 (24.1-203.2)**	**352.9 (80.6->>)**

THI: Tinnitus Handicap Inventory.

[a] ORs were estimated using unconditional multiple logistic regression models after adjustment for sex (men or women), age (<35, 35-44, 45-54, 55-64, 65-74, or ≥75 years), level of education (low or high), and hearing ability (yes, cannot hear at all; yes, severe difficulty; yes, moderate difficulty; yes, a slight difficulty; no difficulty). Estimates in bold are statistically significant at 0.05 level.

Table S2. Sociodemographic and psychological characteristics for participants with tinnitus with or without hyperacusis. Income refers to yearly income in SEK. Pairwise comparisons using Pearson's Chi-square test are reported. Percentages (%) displayed refer to column percentages. P values adjusted for multiple comparisons are shown between parenthesis. Estimates in bold are statistically significant at 0.05 level.

	**Any Tinnitus**		**Severe tinnitus (THI ≥58)**	
Hyperacusis	**Yes (n = 1388)** n (%)	**No (n = 1044)** n (%)	**Yes (n = 156)** n (%)	**No (n = 38)** n (%)
**Sex**	**$\chi^2(1)$=48.5, p<0.0001 (<0.0001)**		$\chi^2(1)$=0.75, p=0.387 (0.635)	
Male	617 (44.5)	613 (58.7)	74 (47.4)	21 (55.3)
Female	771 (55.6)	431 (41.3)	82 (52.6)	17 (44.7)
**Age Group**	$\chi^2(7)$=11.39, p=0.122 (0.122)		$\chi^2(6)$=3.76, p=0.709 (0.851)	
<24	36 (2.6)	20 (1.9)	8 (5.2)	2 (5.3)
25-34	306 (22.1)	186 (17.9)	39 (25.3)	5 (13.2)
35-44	311 (22.5)	228 (21.9)	28 (18.2)	7 (18.4)
45-54	366 (26.4)	286 (27.5)	37 (24)	10 (26.3)
55-64	175 (12.6)	162 (15.6)	22 (14.3)	9 (23.7)
65-74	161 (11.6)	133 (12.8)	15 (9.7)	4 (10.5)
75-84	28 (2)	24 (2.3)	5 (3.3)	1 (2.6)
>85	2 (0.1)	1 (0.1)	0 (0)	0 (0)
**Marital Status**	**$\chi^2(4)$=20.37, p=0.0004 (0.0006)**		$\chi^2(4)$=7.13, p=0.129 (0.543)	
Married	571 (41.1)	481 (46.1)	53 (34)	16 (42.1)
Living with partner	426 (30.7)	311 (29.8)	53 (34)	15 (39.5)
Single	284 (20.5)	151 (14.5)	39 (25)	3 (7.9)
Widow/Widower	18 (1.3)	10 (1)	3 (1.9)	0 (0)
Divorced	89 (6.4)	91 (8.7)	8 (5.1)	4 (10.5)
**Gross income**	**$\chi^2(3)$=46.51, p<0.0001 (<0.0001)**		$\chi^2(3)$=2.8, p=0.424 (0.635)	
0 - 200 000 SEK	204 (14.7)	92 (8.8)	35 (22.4)	8 (21.1)
200 001 - 450 000 SEK	698 (50.3)	488 (46.7)	84 (53.9)	21 (55.3)
450 001 SEK or more	410 (29.5)	428 (41)	22 (14.1)	8 (21.1)
Don't know/don't want to disclose	76 (5.5)	36 (3.5)	15 (9.6)	1 (2.6)
**Education Level**	**$\chi^2(3)$=11.77, p=0.008 (0.01)**		$\chi^2(3)$=4.88, p=0.181 (0.543)	
Middle School	33 (2.4)	30 (2.9)	11 (7.1)	3 (7.9)
High School	276 (19.9)	237 (22.7)	46 (29.5)	15 (39.5)
University	929 (66.9)	703 (67.3)	75 (48.1)	19 (50)
Other	150 (10.8)	74 (7.1)	24 (15.4)	1 (2.6)
**Employment status**	**$\chi^2(10)$=75.25, p<0.0001 (<0.0001)**		$\chi^2(8)$=4.44, p=0.816 (0.816)	
Don't know	1 (0.1)	0 (0)	0 (0)	0 (0)
Employed	836 (60.2)	672 (64.4)	76 (48.7)	21 (55.3)
Unemployed	23 (1.7)	8 (0.8)	7 (4.5)	1 (2.6)
Running my own business/Working as a partner in a company	165 (11.9)	138 (13.2)	11 (7.1)	4 (10.5)
Retired	171 (12.3)	142 (13.6)	20 (12.8)	4 (10.5)
Sick leave (for more than two month) or disability pension due to illness or disability	76 (5.5)	18 (1.7)	23 (14.7)	4 (10.5)
Parental leave (since two months or longer)	20 (1.4)	19 (1.8)	2 (1.3)	1 (2.6)
Student	68 (4.9)	33 (3.2)	15 (9.6)	2 (5.3)
Sabbatical	4 (0.3)	1 (0.1)	0 (0)	0 (0)
Housewife/-Husband	0 (0)	1 (0.1)	0 (0)	0 (0)
Other	24 (1.7)	12 (1.2)	2 (1.3)	1 (2.6)

Table S3. Questionnaire scores from participants with tinnitus with or without hyperacusis. Values are mean (± SD). Pairwise comparisons using Wilcoxon's tests are reported below the compared values. P values adjusted for multiple comparisons are shown between parenthesis. Abbreviations: Numerical Ratins Score (NRS), Tinnitus loudness (Lo), Awareness (Aw), Annoyance (An), Tinnitus Handicap Inventory (THI), Tinnitus Functional Index (TFI), Fear of Tinnitus Questionnaire (FTQ), Tinnitus Catastrophising Scale (TCS), Hyperacusis Questionnaire (HQ), Perceived Stress Questionnaire (PSQ), Hospital Anxiety Depression Scales for Anxiety (HADS A) and depression (HADS D), Quality of Life (QoL) subscales from the World Health Organization: Physical (Phy), Psychological (Psych), Social (Soc), and Environmental (Env). Estimates in bold are statistically significant at 0.05 level.

	**Any Tinnitus**		**Severe tinnitus (THI ≥58)**	
	**Yes (n = 1388)**	**No (n = 1044)**	**Yes (n = 156)**	**No (n = 38)**
Hyperacusis	**Mean (SD)**	**Mean (SD)**	**Mean (SD)**	**Mean (SD)**
NRS Lo	43.7 (25.84)	36.9 (24.3)	74.9 (19.07)	71.5 (22.19)
	**-6.46, p<0.0001 (<0.0001)**		-0.6, p=0.55 (0.872)	
NRS Aw	36.5 (32.41)	28.8 (29.19)	73.8 (26.1)	78.4 (22.45)
	**-5.97, p<0.0001 (<0.0001)**		0.94, p=0.346 (0.872)	
NRS An	23 (27.23)	15.5 (21.9)	65 (26.55)	66.7 (28.65)
	**-8.06, p<0.0001 (<0.0001)**		0.55, p=0.584 (0.872)	
THI	25.8 (22.21)	16.2 (16.67)	73.6 (12.22)	72 (10.18)
	**-12.05, p<0.0001 (<0.0001)**		-0.49, p=0.624 (0.872)	
TFI	25.4 (22.45)	17.1 (17.63)	66.8 (15.91)	65.5 (15.45)
	**-9.48, p<0.0001 (<0.0001)**		-0.42, p=0.676 (0.872)	
PSQ	0.4 (0.18)	0.3 (0.17)	0.5 (0.17)	0.5 (0.21)
	**-10.89, p<0.0001 (<0.0001)**		-0.17, p=0.868 (0.876)	
HADS_A	6.7 (4.3)	5.3 (3.79)	10.6 (4.42)	10.7 (4.74)
	**-8.27, p<0.0001 (<0.0001)**		0.19, p=0.848 (0.876)	
HADS_D	6.7 (4.3)	5.3 (3.79)	10.6 (4.42)	10.7 (4.74)
	**-8.18, p<0.0001 (<0.0001)**		0.65, p=0.519 (0.872)	
FTQ	5.2 (2.83)	4.1 (2.42)	9.5 (2.88)	9.1 (2.46)
	**-10.12, p<0.0001 (<0.0001)**		-1.08, p=0.279 (0.872)	
TCS	14.2 (10.84)	10.4 (9.29)	30.5 (9.26)	31.5 (7.89)
	**-9.28, p<0.0001 (<0.0001)**		0.66, p=0.511 (0.872)	
HQ	20.4 (8.75)	11.9 (7.64)	28.1 (7.4)	20.5 (9.63)
	**-22.72, p<0.0001 (<0.0001)**		**-4.44, p<0.0001 (0.0008)**	
QoL Phy	15.2 (2.83)	16.3 (2.44)	12.4 (2.84)	12.9 (2.83)
	**9.58, p<0.0001 (<0.0001)**		0.96, p=0.338 (0.872)	
QoL Psy	14.4 (2.8)	15.4 (2.4)	11.9 (2.97)	12 (2.49)
	**9.14, p<0.0001 (<0.0001)**		0.39, p=0.697 (0.872)	
QoL Social	13.9 (3.17)	14.5 (2.9)	12.5 (3.43)	12.5 (3.31)
	**4.74, p<0.0001 (<0.0001)**		0.16, p=0.876 (0.876)	
QoL Env	16 (2.37)	16.7 (1.96)	14.2 (2.68)	14.6 (2.64)
	**7.63, p<0.0001 (<0.0001)**		0.75, p=0.452 (0.872)	

Table S4. Phenotypic characteristics for participants with tinnitus with or without hyperacusis. Pairwise comparisons using Pearson's Chi-square test are reported. Percentages (%) displayed refer to column percentages. P values adjusted for multiple comparisons are shown between parenthesis. Estimates in bold are statistically significant at 0.05 level.

	**Any Tinnitus**		**Severe tinnitus (THI ≥58)**	
Hyperacusis	**Yes (n = 1388) n (%)**	**No (n = 1044) n (%)**	**Yes (n = 156) n (%)**	**No (n = 38) n (%)**
**Tinnitus onset**	**$\chi^2(5)$=16.65, p=0.005 (0.007)**		$\chi^2(5)$=6, p=0.306 (0.531)	
Don't know	108 (7.8)	109 (10.4)	4 (2.6)	0 (0)
0 to 6 months	25 (1.8)	25 (2.4)	7 (4.5)	3 (7.9)
6 months to 3 years	195 (14.1)	163 (15.6)	28 (18)	11 (29)
3 to 10 years	382 (27.5)	311 (29.8)	42 (26.9)	6 (15.8)
10 to 20 years	446 (32.1)	267 (25.6)	47 (30.1)	9 (23.7)
More than 20 years	232 (16.7)	169 (16.2)	28 (18)	9 (23.7)
**Onset-related events**	**$\chi^2(6)$=30.74, p<0.0001 (<0.0001)**		$\chi^2(6)$=4.74, p=0.578 (0.753)	
Loud blast of sound	581 (41.9)	383 (36.7)	50 (32.1)	9 (23.7)
Stress	198 (14.3)	132 (12.6)	24 (15.4)	10 (26.3)
Change in hearing	89 (6.4)	45 (4.3)	18 (11.5)	3 (7.9)
Head trauma	10 (0.7)	8 (0.8)	1 (0.6)	0 (0)
Whiplash	11 (0.8)	8 (0.8)	1 (0.6)	0 (0)
Other	190 (13.7)	136 (13)	32 (20.5)	6 (15.8)
Don't know	309 (22.3)	332 (31.8)	30 (19.2)	10 (26.3)
**Tinnitus occurrence**	**$\chi^2(1)$=17.01, p<0.0001 (<0.0001)**		$\chi^2(1)$=1.25, p=0.263 (0.531)	
Occasionally (now and then)	559 (40.3)	508 (48.7)	12 (7.7)	1 (2.6)
Always (all the time)	829 (59.7)	536 (51.3)	144 (92.3)	37 (97.4)
**Time of the day of tinnitus emergence**	**$\chi^2(6)$=17.94, p=0.006 (0.008)**		$\chi^2(6)$=3.26, p=0.775 (0.894)	
Don't know	716 (51.6)	582 (55.8)	50 (32.1)	13 (34.2)
When awakening	108 (7.8)	46 (4.4)	28 (18)	5 (13.2)
In the morning	24 (1.7)	24 (2.3)	4 (2.6)	3 (7.9)
Around noon	91 (6.6)	67 (6.4)	18 (11.5)	5 (13.2)
In the afternoon	70 (5)	41 (3.9)	16 (10.3)	4 (10.5)
In the evening	183 (13.2)	119 (11.4)	28 (18)	6 (15.8)
Before sleeping	196 (14.1)	165 (15.8)	12 (7.7)	2 (5.3)
**Perceiving the onset of tinnitus**	**$\chi^2(2)$=9.63, p=0.008 (0.01)**		$\chi^2(2)$=4.87, p=0.088 (0.435)	
Don't know	329 (23.7)	302 (28.9)	10 (6.4)	5 (13.2)
Gradual	608 (43.8)	445 (42.6)	64 (41)	9 (23.7)
Abrupt	451 (32.5)	297 (28.5)	82 (52.6)	24 (63.2)
**Pulsatility**	**$\chi^2(3)$=17.94, p=0.001 (0.001)**		$\chi^2(3)$=1.6, p=0.659 (0.808)	
Don't know	124 (8.9)	70 (6.7)	11 (7.1)	5 (13.2)
Yes, with heart beat	113 (8.1)	61 (5.8)	20 (12.8)	4 (10.5)
Yes, different from heart beat	66 (4.8)	28 (2.7)	19 (12.2)	4 (10.5)
No	1085 (78.2)	885 (84.8)	106 (68)	25 (65.8)
**Location of tinnitus**	$\chi^2(6)$=12.6, p=0.05 (0.053)		$\chi^2(6)$=5.64, p=0.465 (0.697)	
Right ear	117 (8.4)	72 (6.9)	13 (8.3)	3 (7.9)
Left ear	108 (7.8)	102 (9.8)	15 (9.6)	7 (18.4)
Both ears, worse in right	266 (19.2)	164 (15.7)	29 (18.6)	7 (18.4)
Both ears, worse in left	223 (16.1)	180 (17.2)	37 (23.7)	5 (13.2)
Both ears equally	443 (31.9)	354 (33.9)	27 (17.3)	10 (26.3)
Inside the head	212 (15.3)	165 (15.8)	28 (18)	5 (13.2)

Elsewhere	19 (1.4)	7 (0.7)	7 (4.5)	1 (2.6)
**Sound of tinnitus**	**$\chi^2(9)$=27.5, p=0.001 (0.002)**		$\chi^2(7)$=8.74, p=0.272 (0.531)	
Tone	256 (18.6)	255 (24.6)	19 (12.5)	3 (7.9)
Noise	155 (11.3)	115 (11.1)	11 (7.2)	4 (10.5)
Crickets	43 (3.1)	42 (4.1)	5 (3.3)	2 (5.3)
Heartbeat	5 (0.4)	6 (0.6)	2 (1.3)	0 (0)
Beeping	152 (11.1)	136 (13.1)	12 (7.9)	8 (21.1)
Morse Code	2 (0.2)	1 (0.1)	0 (0)	0 (0)
An alarm	11 (0.8)	8 (0.8)	4 (2.6)	1 (2.6)
Other	44 (3.2)	21 (2)	7 (4.6)	3 (7.9)
Don't know	0 (0)	2 (0.2)	0 (0)	0 (0)
Complex	706 (51.4)	451 (43.5)	92 (60.5)	17 (44.7)
**Tinnitus loudness variation from day to day**	$\chi^2(5)$=25.38, p=0.0001 (0.0002)		$\chi^2(5)$=6.58, p=0.254 (0.531)	
Don't know	78 (5.6)	76 (7.3)	3 (1.9)	0 (0)
Never	88 (6.3)	96 (9.2)	7 (4.5)	5 (13.2)
Seldom	183 (13.2)	184 (17.6)	18 (11.5)	7 (18.4)
Sometimes	549 (39.6)	358 (34.3)	67 (43)	12 (31.6)
Often	341 (24.6)	215 (20.6)	39 (25)	9 (23.7)
Always	149 (10.7)	115 (11)	22 (14.1)	5 (13.2)
**Pitch of tinnitus**	$\chi^2(4)$=5.75, p=0.218 (0.218)		$\chi^2(4)$=5.26, p=0.262 (0.531)	
Don't know	59 (4.3)	35 (3.4)	2 (1.3)	2 (5.3)
Very high frequency	347 (25)	244 (23.4)	55 (35.3)	15 (39.5)
High frequency	625 (45)	462 (44.3)	63 (40.4)	13 (34.2)
Medium frequency	261 (18.8)	209 (20)	28 (18)	4 (10.5)
Low frequency	96 (6.9)	94 (9)	8 (5.1)	4 (10.5)
**Reduction of tinnitus by music or environmental sounds**	**$\chi^2(2)$=10.21, p=0.006 (0.008)**		$\chi^2(2)$=1.27, p=0.53 (0.731)	
Don't know	324 (23.3)	282 (27)	19 (12.2)	6 (15.8)
Yes	782 (56.3)	598 (57.3)	86 (55.1)	23 (60.5)
No	282 (20.3)	164 (15.7)	51 (32.7)	9 (23.7)
**Worsening of tinnitus by loud noise**	**$\chi^2(2)$=197.21, p<0.0001 (<0.0001)**		**$\chi^2(2)$=18.84, p<0.0001 (<0.0001)**	
Don't know	352 (25.4)	303 (29)	35 (22.4)	8 (21.1)
Yes	838 (60.4)	367 (35.2)	104 (66.7)	15 (39.5)
No	198 (14.3)	374 (35.8)	17 (10.9)	15 (39.5)
**Tinnitus affected by head movement or touch**	**$\chi^2(2)$=16.53, p=0.0003 (0.0005)**		$\chi^2(2)$=3.48, p=0.176 (0.527)	
Don't know	289 (20.8)	203 (19.4)	15 (9.6)	4 (10.5)
Yes	387 (27.9)	225 (21.6)	83 (53.2)	14 (36.8)
No	712 (51.3)	616 (59)	58 (37.2)	20 (52.6)
**Tinnitus affected by nap**	**$\chi^2(3)$=8.3, p=0.04 (0.045)**		$\chi^2(3)$=3.52, p=0.319 (0.531)	
Don't know	795 (57.3)	587 (56.2)	54 (34.6)	13 (34.2)
It mainly worsens my tinnitus	39 (2.8)	14 (1.3)	19 (12.2)	2 (5.3)
It mainly reduces my tinnitus	435 (31.3)	361 (34.6)	71 (45.5)	17 (44.7)
It has no effect	119 (8.6)	82 (7.9)	12 (7.7)	6 (15.8)
**Tinnitus affected by bad nights sleep**	**$\chi^2(5)$=54.02, p<0.0001 (<0.0001)**		$\chi^2(5)$=1.39, p=0.925 (0.957)	
Don't know	506 (36.5)	414 (39.7)	22 (14.1)	7 (18.4)
Never	165 (11.9)	195 (18.7)	11 (7.1)	3 (7.9)
Seldom	102 (7.4)	91 (8.7)	14 (9)	4 (10.5)
Sometimes	325 (23.4)	218 (20.9)	40 (25.6)	7 (18.4)

Often	215 (15.5)	105 (10.1)	45 (28.9)	10 (26.3)
Always	75 (5.4)	21 (2)	24 (15.4)	7 (18.4)
**Tinnitus affected by stress**	**$\chi^2(3)=59.96$, p<0.0001 (<0.0001)**		$\chi^2(2)=2.41$, p=0.299 (0.531)	
Don't know	469 (33.8)	382 (36.6)	25 (16)	6 (15.8)
Yes, it worsens my tinnitus	675 (48.6)	364 (34.9)	119 (76.3)	26 (68.4)
Yes, it reduces my tinnitus	242 (17.4)	295 (28.3)	12 (7.7)	6 (15.8)
No, it has no effect	2 (0.1)	3 (0.3)	0 (0)	0 (0)
**Tinnitus affected by medication**	**$\chi^2(2)=11.11$, p=0.004 (0.006)**		$\chi^2(2)=0.19$, p=0.91 (0.957)	
Don't know	1038 (74.8)	745 (71.4)	96 (61.5)	22 (57.9)
Yes	43 (3.1)	18 (1.7)	16 (10.3)	4 (10.5)
No	307 (22.1)	281 (26.9)	44 (28.2)	12 (31.6)
**Contacted a clinician due to tinnitus**	**$\chi^2(2)=45.07$, p<0.0001 (<0.0001)**		$\chi^2(2)=1.25$, p=0.536 (0.731)	
No	810 (58.4)	745 (71.4)	23 (14.7)	7 (18.4)
Yes, because of curiosity	77 (5.6)	49 (4.7)	4 (2.6)	0 (0)
Yes, because I sought for help	501 (36.1)	250 (24)	129 (82.7)	31 (81.6)
**Number of tinnitus treatments**	**$\chi^2(3)=24.14$, p<0.0001 (<0.0001)**		$\chi^2(3)=6.22$, p=0.102 (0.435)	
None	1177 (84.8)	949 (90.9)	87 (55.8)	17 (44.7)
1	86 (6.2)	47 (4.5)	21 (13.5)	8 (21.1)
2-4	86 (6.2)	39 (3.7)	26 (16.7)	11 (29)
5 or more	39 (2.8)	9 (0.9)	22 (14.1)	2 (5.3)
**Tinnitus occurence in family**	**$\chi^2(1)=24.59$, p<0.0001 (<0.0001)**		$\chi^2(1)=3.41$, p=0.065 (0.435)	
No	1086 (78.2)	899 (86.1)	108 (69.2)	32 (84.2)
Yes	302 (21.8)	145 (13.9)	48 (30.8)	6 (15.8)

Table S5. Comorbidities in participants with tinnitus with or without hyperacusis. Pairwise comparisons using Pearson's Chi-square test are reported. Percentages (%) displayed refer to column percentages. P values adjusted for multiple comparisons are shown between parenthesis. Estimates in bold are statistically significant at 0.05 level.

	Any Tinnitus		Severe tinnitus (THI ≥58)	
Hyperacusis	**Yes (n = 1388)** **n (%)**	**No (n = 1044)** **n (%)**	**Yes (n = 156)** **n (%)**	**No (n = 38)** **n (%)**
**Hearing problem**	**$\chi^2(2)$=7.16, p=0.028 (0.032)**		$\chi^2(2)$=3.48, p=0.176 (0.527)	
Don't know	226 (16.3)	167 (16)	13 (8.3)	4 (10.5)
Yes	720 (51.9)	492 (47.1)	103 (66)	19 (50)
No	442 (31.8)	385 (36.9)	40 (25.6)	15 (39.5)
**Hearing aids**	**$\chi^2(3)$=9.35, p=0.025 (0.03)**		$\chi^2(3)$=3.67, p=0.3 (0.531)	
Yes, on both ears	101 (7.3)	47 (4.5)	25 (16)	7 (18.4)
Yes, on the right ear	14 (1)	7 (0.7)	4 (2.6)	2 (5.3)
Yes, on the left ear	18 (1.3)	11 (1.1)	4 (2.6)	3 (7.9)
No	1255 (90.4)	979 (93.8)	123 (78.9)	26 (68.4)
**Problems tolerating sounds**	**$\chi^2(4)$=685.97, p<0.0001 (<0.0001)**		**$\chi^2(3)$=18.36, p=0 (0.006)**	
Never	4 (0.3)	103 (9.9)	0 (0)	0 (0)
Rarely	62 (4.5)	328 (31.4)	4 (2.6)	6 (15.8)
Sometimes	483 (34.8)	437 (41.9)	29 (18.6)	13 (34.2)
Usually	517 (37.3)	141 (13.5)	53 (34)	11 (29)
Always	322 (23.2)	35 (3.4)	70 (44.9)	8 (21.1)
**Headache**	**$\chi^2(2)$=72.45, p<0.0001 (<0.0001)**		$\chi^2(2)$=1.77, p=0.413 (0.652)	
Don't know	42 (3)	5 (0.5)	6 (3.9)	0 (0)
Yes	427 (30.8)	194 (18.6)	62 (39.7)	14 (36.8)
No	919 (66.2)	845 (80.9)	88 (56.4)	24 (63.2)
**Temporomandibular problems**	**$\chi^2(2)$=73.27, p<0.0001 (<0.0001)**		$\chi^2(2)$=5.14, p=0.077 (0.435)	
Don't know	73 (5.3)	22 (2.1)	10 (6.4)	3 (7.9)
Yes	326 (23.5)	126 (12.1)	59 (37.8)	7 (18.4)
No	989 (71.3)	896 (85.8)	87 (55.8)	28 (73.7)
**Vertigo/dizziness**	**$\chi^2(2)$=45.72, p<0.0001 (<0.0001)**		$\chi^2(2)$=9.03, p=0.011 (0.109)	
Don't know	70 (5)	26 (2.5)	6 (3.9)	0 (0)
Yes	381 (27.5)	186 (17.8)	54 (34.6)	5 (13.2)
No	937 (67.5)	832 (79.7)	96 (61.5)	33 (86.8)
**Neck pain**	**$\chi^2(2)$=42.03, p<0.0001 (<0.0001)**		$\chi^2(2)$=0.79, p=0.673 (0.808)	
Don't know	27 (2)	13 (1.3)	2 (1.3)	1 (2.6)
Yes	514 (37)	263 (25.2)	80 (51.3)	17 (44.7)
No	847 (61)	768 (73.6)	74 (47.4)	20 (52.6)
**Other pain syndromes**	**$\chi^2(2)$=34.65, p<0.0001 (<0.0001)**		$\chi^2(2)$=4.02, p=0.134 (0.503)	
Don't know	25 (1.8)	13 (1.3)	4 (2.6)	2 (5.3)
Yes	386 (27.8)	187 (17.9)	63 (40.4)	9 (23.7)
No	977 (70.4)	844 (80.8)	89 (57.1)	27 (71.1)
**Under psychatric treatment**	**$\chi^2(2)$=32.91, p<0.0001 (<0.0001)**		$\chi^2(1)$=0.01, p=0.909 (0.957)	
Don't know	6 (0.4)	9 (0.9)	0 (0)	0 (0)
Yes	157 (11.3)	51 (4.9)	30 (19.2)	7 (18.4)
No	1225 (88.3)	984 (94.3)	126 (80.8)	31 (81.6)
**Diagnosed disease**	$\chi^2(1)$=2.73, p=0.098 (0.102)		$\chi^2(1)$=0.001, p=0.969 (0.969)	
Yes	479 (34.5)	327 (31.3)	58 (37.2)	14 (36.8)
No	909 (65.5)	717 (68.7)	98 (62.8)	24 (63.2)
